# The Association Between Dietary Antioxidant Micronutrients and Cardiovascular Disease in Adults in the United States: A Cross-Sectional Study

**DOI:** 10.3389/fnut.2021.799095

**Published:** 2022-01-12

**Authors:** Ting Yin, Xu Zhu, Dong Xu, Huapeng Lin, Xinyi Lu, Yuan Tang, Mengsha Shi, Wenming Yao, Yanli Zhou, Haifeng Zhang, Xinli Li

**Affiliations:** ^1^Department of Cardiology, The First Affiliated Hospital of Nanjing Medical University, Jiangsu Province Hospital, Nanjing, China; ^2^Department of Vascular Surgery, Affiliated Hangzhou First People's Hospital, Zhejiang University School of Medicine, Zhejiang, China; ^3^Department of Medicine and Therapeutics, The Chinese University of Hong Kong, Hong Kong, Hong Kong SAR, China; ^4^Department of Cardiology, The Affiliated Suzhou Hospital of Nanjing Medical University, Suzhou Municipal Hospital, Gusu School, Nanjing Medical University, Suzhou, China

**Keywords:** antioxidant micronutrients, dietary nutrient intake, cardiovascular disease, disease nutrition interaction, weight quantile sum, restricted cubic spline, US adults

## Abstract

**Background:** Antioxidant micronutrients represent an important therapeutic option for the treatment of oxidative stress-associated cardiovascular diseases (CVDs). However, few studies have evaluated the relationship between the levels of multiple dietary antioxidants and CVDs.

**Objective:** The study therefore aimed to evaluate associations between dietary antioxidants and total and specific CVDs among a nationally representative sample of adults in the US.

**Design:** In total, 39,757 adults (>20 years) were included in this cross-sectional study from the 2005–2018 National Health and Nutrition Examination Survey. We analyzed dietary recall of 11 antioxidant micronutrients in this population. Multivariate logistic and weighted quantile sum (WQS) regression were both applied to examine the relationships between these antioxidants, alone and in combination, with the prevalence of all CVDs and specific CVDs. The linearity of these correlations was also explored using restricted cubic spline (RCS) regression.

**Results:** Multivariate logistic models showed that, compared with the lowest quartile, the levels of 11 antioxidants in the highest quartile were independently associated with decreased total CVD (all *P* < 0.05). The WQS index showed that, when considered together, the 11 micronutrients were negatively correlated with total CVD (*P* < 0.001) and five specific CVDs (all *P* < 0.05), and selenium had the strongest association (weight = 0.219) with total CVD. Moreover, the RCS model demonstrated that iron, zinc and copper were all negatively and non-linearly correlated with total CVD, while the eight other micronutrients had non-significant, linear, negative relationships with total CVD (*P* for non-linearity >0.05). A piecewise binary logistic regression analysis showed that the inflection points in the relationships between CVD and iron, zinc and copper were 7.71, 6.61, and 0.74 mg/day, respectively.

**Conclusions:** Our findings suggested that high levels of combined dietary antioxidant micronutrients are associated with decreased prevalence of CVDs, and that selenium has the greatest contribution to this association.

## Highlights

- We analyzed 11 dietary antioxidant micronutrients in collaboration with total cardiovascular disease (CVD) and specific CVDs in a nationally representative United States population.
- Dietary intake of 11 antioxidant micronutrients were independently associated with decreased odds of total CVD and specific CVDs.
- Weighted quantile sum (WQS) analysis showed that, when considered together, the 11 antioxidants were negatively associated with the odds of total CVD and specific CVDs; of all components, selenium was most strongly associated with total CVD.
- Iron, zinc and copper were all non-linearly, negatively correlated with total CVD; inflection points were at 7.71, 6.61, and 0.74 mg/day, respectively.

## Introduction

Cardiovascular disease (CVD) is the leading cause of death and disability in both developed and developing countries, and has become a serious public health problem (1, 2). Numerous studies have been published on CVD risk factors in recent decades; these have established that aging, smoking, obesity, cholesterol levels, poor dietary habits, educational level, blood pressure, diabetes, and genetics, all affect risk (3). At-risk populations would benefit from the effective management of these risk factors. Strikingly, 43.9% of the US adult population is still projected to have some form of CVD by 2030 (4). Epidemiological studies have also shown that 75% of pre-mature CVDs are preventable through early intervention (5). A more comprehensive understanding of CVD etiology and its underlying mechanisms remain a priority.

Oxidative stress plays an important role in the progression of various CVDs, including atherosclerosis, heart failure, cardiac arrhythmia, and ischemia-reperfusion injury (6, 7). Increased oxidative stress can modify DNA and proteins, induce subcellular remodeling and Ca2^+^-handling abnormalities, and lead to functional hypoxia and disordered metabolism; this can result in cellular inflammation and programmed cell death, necrosis, and fibrosis, which are tightly regulated by reactive oxygen species (ROS) production and intracellular defense mechanisms (8, 9). There are a number of therapeutic options available for the treatment of oxidative stress-associated CVDs. Among these, micronutrients are critical for every stage of the antioxidant response. Fundamental studies on the properties of antioxidant micronutrients have shown that the fat-soluble vitamins A (including retinol, carotene and cryptoxanthin) and E (α-tocopherol) and the water-soluble vitamin C (ascorbic acid) all have anti-inflammatory properties (10). These vitamins interact with free radicals and reactive nitrogen species (RNS), decreasing polyunsaturated fatty acid (PUFA) peroxidation and thus protecting cell membrane phospholipids and plasma lipoproteins, with beneficial effects on glutathione (GSH) status and oxidation defense capabilities (11). Similarly, increasing evidence indicates that essential metal micronutrients, such as selenium, zinc, copper, and iron, play critical roles in a wide range of physiological processes; they are integral to the enzymatic system involved in the reduction of oxygen free radicals (12). It is possible that inadequate intake of these nutrients may result in increased ROS, which have an implied role in the mechanisms and risk of CVD.

Observational studies and clinical trials have evaluated the potential role of antioxidant micronutrients and their safe dose for CVD prevention or treatment (13). Epidemiological studies of individual micronutrients reported that dietary vitamin A, carotenoids (14, 15), vitamin C (16, 17), vitamin E (18), selenium (19, 20), zinc (21), iron (22), and copper (23) were associated with lower CVD risk and reduced cardiovascular mortality. These correlations were even more pronounced in the deficiency state of subsequent complement micronutrients and the level of dietary metal micronutrients are closely related to serum concentrations. The metabolism and distribution of various trace vitamins and minerals also alter cellular ion contents, which in turn modulates the metabolic functions of macronutrients (carbohydrates, lipids, and proteins) and levels of probiotic bacteria; this affects intrinsic pathological mechanisms of CVD and cumulative drug therapeutic effects in people with this disease (22), and thus impacts long-term pharmacotherapy.

Notably, the effect of antioxidant micronutrients, alone or in combination, in the prevention or reduction of CVD is controversial. Analyses from 15 trials reporting data on 188,209 participants showed that supplementation of the antioxidant vitamins E and C and β-carotene has no effect on the incidence of major cardiovascular events, myocardial infarction, stroke, total deaths, and cardiac-related deaths (24). Other studies have even suggested that supplementation of antioxidants may increase CVD risk owing to potential peroxidation (23). There are several challenges to overcome in studies on the impact of dietary antioxidants relating to dosage, duration of intervention, and baseline micronutrient status of those receiving interventions, in particular, the interactive effects between vitamins cannot be ignored (25). There is a rapidly growing interest in the health effects of dietary exposure to combinations of dietary antioxidant micronutrients because mixed exposure better represents the mixed diets that people eat in real-world situations. Studies on micronutrient status and the effects of combined supplementation are therefore worthy of further study, but they pose unique challenges to inference (26, 27); few studies have been conducted in this domain. Statistical techniques for analyzing exposure to combined factors have been developed, including weighted quantile sum (WQS), which permits the assessment of the overall effect of combined treatments on health outcomes, and crucially, the weighted contributions of each component used to answer questions about projecting exposures to a higher dimensional space (28).

The National Health and Nutrition Examination Survey (NHANES), which collects detailed data on diet, nutritional status, and chronic disease to inform nutrition and health policy, is the cornerstone for national nutrition monitoring in the US (29). The objectives of the current study were to present an analysis using this large, cross-sectional, nationally-representative database to assess the intake status of v antioxidant micronutrients with a known role in CVD, and explore the effects of combined exposure to these antioxidants on CVD.

## Methods

### Study and Population

This study used data from NHANES, a nationally-representative, cross-sectional database on civilian, non-institutionalized persons living in the United States, administered by the National Center for Health Statistics (NCHS) at the Centers for Disease Control and Prevention. NHANES surveys are demographically based, with samples selected through a complex, multistage survey design (30). A detailed sample design is available at: https://wwwn.cdc.gov/nchs/nhanes/tutorials/module2.aspx). Specifically, we used the NHANES 2003–2018 continuous survey. The data in this survey were collected using a series of large, complex, stratified, multistage probability samples with a 4-year design, with data released in 2-year cycles. Our analysis used data on dietary recall of vitamin intake, collected from the What We Eat in America (WWEIA) component of the NHANES, which is conducted by a partnership between the US Department of Agriculture (USDA) and the US Department of Health and Human Services (DHHS) (31).

Participants provided written informed consent and all study procedures were approved by the National Center for Health Statistics Research Ethics Review Board. None of the authors of this study have ever been involved in the collection or production of the NHANES database. Participants with fewer than two valid 24-h dietary recalls, and those who were younger than 20 years of age for whom data on CVD outcomes were missing, were excluded from our analyses during the 2003–2018 period.

### Measurement of Two 24-h Diet Recalls

Dietary intake data for all participants were used to estimate the types and amounts of foods and beverages (including all types of lipids volume) consumed during the 24-h period prior to a dietary recall interview (midnight to midnight), and to estimate intakes for energy, nutrients, and other food components. All NHANES participants were eligible for two 24-h dietary recall interviews; the first was collected in person in a mobile examination center and the second was collected by telephone, 3–10 days after the first 24-h recall period (30). In the dietary recall investigation in NHANES 2003–2018, two dietary interviews were conducted with all sample members.

Data on intake for 11 antioxidant related micronutrients [vitamin C (DRTVC), iron (DRTIRON); vitamin E as α-tocopherol (DRTATOC); zinc (DRTZINC); β-carotene (DRTBCAR); copper (DRTCOPP); α-carotene (DRTACAR), vitamin A (DRTVARA); retinol (DRTRET); β-cryptoxanthin (DRTCRYP); selenium (DRTSELE)] were included in our analysis. (https://wwwn.cdc.gov/Nchs/Nhanes/2017-2018/DR1IFF_J.htm) (https://wwwn.cdc.gov/Nchs/Nhanes/2017-2018/DR2IFF_J.htm#Appendix_2). The average of two 24-h dietary recall values for each antioxidant in each 2-year cycle from the 16 years of available data was analyzed. These amounts reflected only nutrients obtained from foods, beverages, and water, including tap and bottled water (32); they did not include nutrients obtained from dietary supplements, antacids, or medications.

### CVD Definition

Participants aged ≥20 years old were asked: “Has a doctor or other health professional ever told you that you have X,” where X was congestive heart failure (CHF), coronary heart disease (CHD), angina, heart attack, or stroke. The CVD data in the NHANES 2003–2018 survey was obtained from medical records, laboratory data, and questionnaires. In addition, the data for each CVD outcome were gathered for further analysis of their association with dietary antioxidant micronutrients.

### Covariates

Potential confounding variables for the CVD outcome measures were collected. Sociodemographic characteristics included age in years (modeled continuously), sex (male or female), race/ethnicity (“non-Hispanic white,” “non-Hispanic black,” “Mexican American,” “other Hispanic,” and “other,” including multi-racial), educational level (above high school, high school, and below high school), working status, and poverty status. Health-related covariates included body mass index (BMI; kg/m^2^); smoking status, as defined by responses to two questions (“Have you smoked at least 100 cigarettes during your entire life?”) (https://wwwn.cdc.gov/Nchs/Nhanes/2017-2018/SMQ_J.htm); and habitual drinking status, in which someone was defined as drinking if they answered “yes” to “In your entire life, have you had at least 12 drinks of any kind of alcoholic beverage?” and “In the past 12 months did you have at least 12 drinks of any kind of alcoholic beverage?” (https://wwwn.cdc.gov/Nchs/Nhanes/2017-2018/ALQ_J.htm). We also collected data on comorbidities, including diabetes mellitus and hypertension, as determined through a questionnaire administered by NHANES personnel (Subcommittee of Professional and Public Education of the American Heart Association Council) (https://wwwn.cdc.gov/Nchs/Nhanes/2017-2018/DIQ_J.htm); in addition, dietary supplement intake was recorded in another questionnaire (https://wwwn.cdc.gov/nchs/nhanes/Search/default.aspx.) (33).

### Statistical Analyses

Eleven antioxidant micronutrients were analyzed for their association with CVD overall and with CHD, CHF, heart attack, stroke and angina separately. Normality of continuous variables was assessed using Kolmogorov–Smirnov-tests. Continuous variables were expressed as mean [standard deviation (SD)] and were compared using unpaired *t*-tests. Categorical or dichotomous variables were expressed as absolute value (percentage) and were compared using *χ*^2^-tests. The correlation coefficients for all antioxidant micronutrient dietary intakes were calculated using the Pearson correlation method. The metabolites of antioxidant micronutrients were divided into quartiles, and the lowest quartile was used as a reference category. Concentrations of each of the 11 micronutrients were log-transformed to normalize their distributions for further analysis.

Multivariate logistic regression models were used to calculate odds ratios (ORs) and 95% confidence intervals (CIs) to assess the aggregate specific CVDs prevalence associated with each of the 11 micronutrients. Three models were used, with increasing levels of adjustment for confounding variables: in model 1, data were adjusted for age and sex; model 2 was based on model 1, with additional adjustments for race, education levels and poverty; and model 3 was based on model 2, with additional adjustments for smoking, drinking, BMI, total cholesterol, dietary supplement use, diabetes, and hypertension.

Because of high correlations between dietary intake values for various vitamins, we performed WQS regression using the gWQS in R v.3.6.1 (34). These analyses were used to assess associations between the level of all antioxidant micronutrients in combination and CVD, and to evaluate these as predictors of CVD in logistic regression models. Each micronutrient was assigned a weight within the index that indicates its contribution to the overall association (35). In the gWQS function, we used deciles for exposure weighting, 1,000 bootstrap repetitions, a random seed set to 2018, and a binomial distribution for the general linear model. A constraint of this approach is that, in each case, weights are estimated by pooling effects only in the positive or the negative direction. Individual vitamin weights of ≥0.1 were considered significant contribution rate. The value of 0.1 was chosen for ease of comparison across models that included different numbers of antioxidants.

The shape of the relationship between dietary micronutrient intake and risk of CVD were explored using the restricted cubic spline (RCS) regression model with three knots (10th, 50th, and 90th) percentile of antioxidants and analysis of variance (ANOVA) was used to test for non-linearity (36). If non-linearity was detected, segmented regression was used to fit the piecewise-linear relationship between micronutrients and total CVD or specific CVDs, and to calculate the threshold inflection point using a recursive algorithm, as described previously (37). The significance of each interaction (*P* interaction) was tested using the likelihood ratio test. Significance was set to *P* < 0.05 (two-sided).

## Results

### Study Population Characteristics According to Total CVD Status

**Figure 1:**
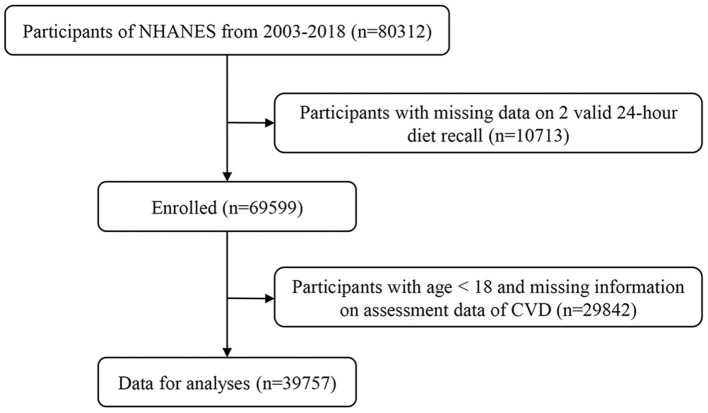
Study flow diagram.

In total, 80,312 people were initially enrolled. Ten thousand seven hundred and thirteen participants with fewer than two valid 24-h dietary recalls and 29,842 people younger than 20 years of age with missing data on CVD outcomes were excluded, leaving a total of 39,757 participants who were included in our analyses (Figure 1), comprising 19,279 (48.5%) male and 20,460 (47.5%) female participants, with a mean age of 49.6 ± 18.0 years old. For the purposes of our analysis, we separated these into those without CVD (*n* = 35,280, 47.5%) and those with CVD (*n* = 4,477, 56.6%). Table 1 shows the demographic characteristics of these two groups. Participants with CVD were generally older (66.7 ± 13.0 vs. 47.5 ± 17.4), comprising a greater proportion of men (56.6 vs. 47.5%), more high-school educated individuals (25.3 vs. 23.0%), more non-Hispanic participants (55.5 vs. 42.5%), higher poverty states (23.1 vs. 21.6%), a greater proportion of smokers (61.2 vs. 43.3%), higher BMI (7.0 vs. 6.8%), greater dietary supplement use (59.1 vs. 49.9%), and greater rates of diabetes (33.0 vs. 10.1%) and hypertension (73.4 vs. 31.1%).These differences between the two groups were statistically significant (all *P* < 0.05).

**Table 1:** Sociodemographic characteristics of the study population.

**Variable**	**Total** **(***n*** = 39,757)**	**Non-CVD** **(***n*** = 35,280)**	**CVD** **(***n*** = 4,477)**	***P*** **-value**
Age, years, Mean ± SD	49.61 ± 18.04	47.46 ± 17.43	66.73 ± 12.9	<0.001
Male, *N* (%)	19,279 (48.49%)	16,746 (47.47%)	2,533 (56.58%)	<0.001
Education level, *N* (%)				<0.001
Below high school	10,033 (25.24%)	8,524 (24.16%)	1,509 (33.71%)	
High school	9,264 (23.30%)	8,131 (23.05%)	1,133 (25.31%)	
Above high school	20,460 (51.46%)	18,625 (52.79%)	1,835 (40.98%)	
Race/ethnicity, *N* (%)				<0.001
Mexican American	6,413 (16.13%)	5,948 (16.89%)	465 (10.39%)	
Other Hispanic	3,482 (9.66%)	3,193 (9.05%)	289 (6.45%)	
Non-Hispanic White	17,481 (43.97 %)	14,996 (42.51%)	2,485 (55.51%)	
Non-Hispanic Black	8,511 (21.41%)	7,546 (21.39%)	965 (21.55%)	
Other race	3,870 (9.73%)	3,597 (10.20%)	273 (6.10%)	
Poverty, *N* (%)	8,315 (20.91%)	7,280 (20.63%)	1,035 (23.12%)	0.007
Smoker, *N* (%)	18,021 (45.32%)	15,281 (43.31%)	2,740 (61.20%)	<0.001
Drinking, *N* (%)	27,962 (70.33%)	24,947 (70.71%)	3,015 (67.34%)	
Body mass index, kg/m^2^, Mean ± SD	29.15 ± 6.87	29.04 ± 6.77	30.33 ± 7.29	<0.001
Total cholesterol, mmol/L, Mean ± SD	4.86 ± 1.13	5.14 ± 1.13	4.72 ± 1.07	<0.001
Dietary supplement use, *N* (%)	20,241 (50.91%)	17,596 (49.88%)	2,645 (59.08%)	<0.001
Diabetes, *N* (%)	5,039 (12.67%)	3,562 (10.10%)	1,477 (32.99%)	<0.001
Hypertension, *N* (%)	14,242 (35.82%)	10,956 (31.05 %)	3,286 (73.40%)	<0.001

*CVD, cardiovascular disease*.

*Data are presented as mean (SD) or median (interquartile range), or n (%)*.

### Distribution of and Correlation Between Antioxidant Micronutrient Levels

Overall, Supplementary Table S1 shows the detected concentrations and distribution of the 11 antioxidant micronutrients, with the highest levels observed for vitamin C, followed by iron, vitamin E, zinc, β-carotene, copper, α-carotene, vitamin A, retinol, β-cryptoxanthin, and lowest levels observed for selenium.

**Figure 2:**
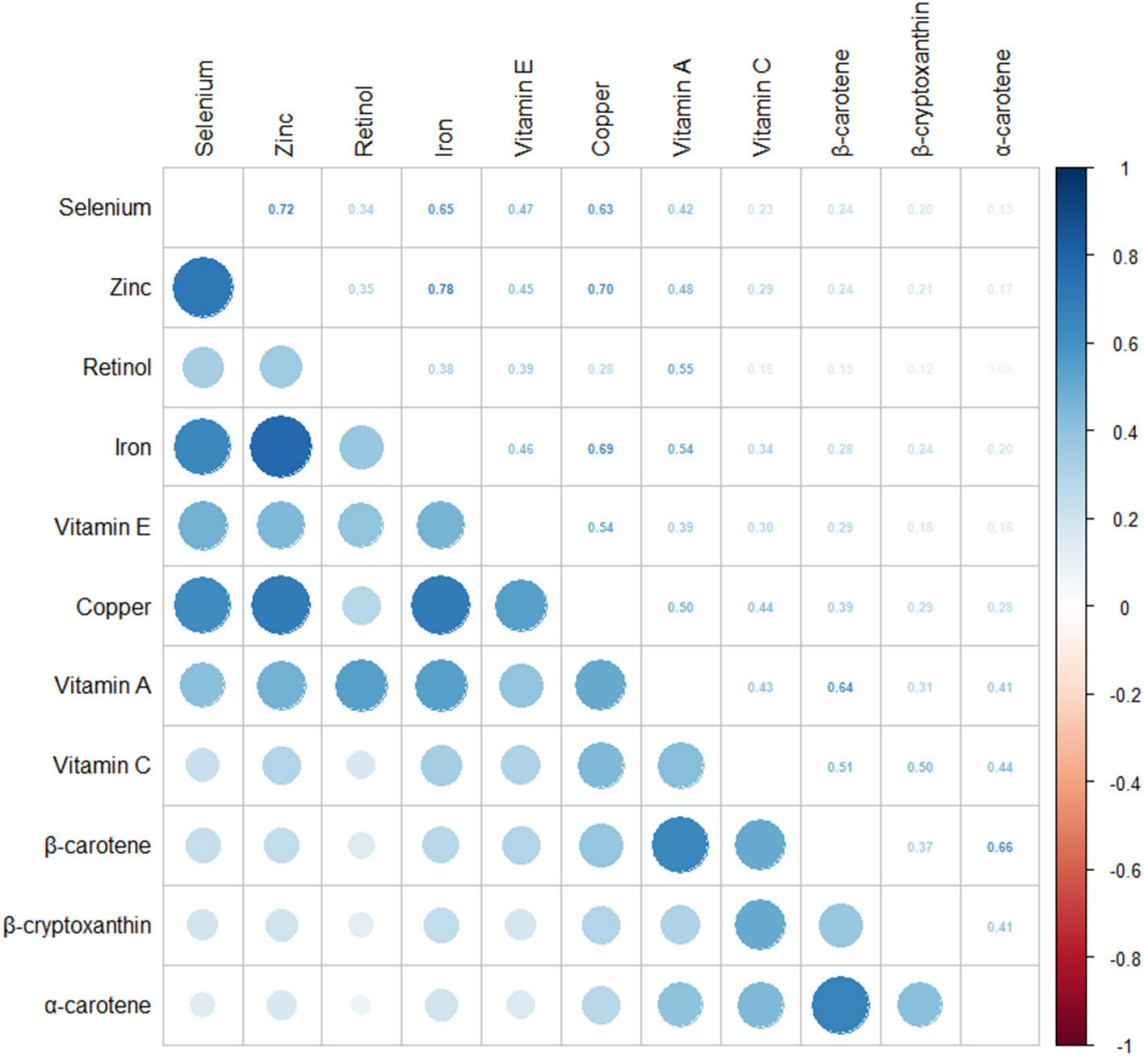
Pairwise Pearson correlation coefficients between dietary intake of 11 antioxidant micronutrients in adults from the United States, collected from the National Health and Nutritional Examination Survey (NHANES) database 2003–2018.

Correlation analyses showed that most of the antioxidant micronutrients were moderately correlated with the other 10 vitamins (Spearman's rank *r* ≥ 0.3). An r value of ≥0.7 was found in the correlations between zinc and iron, α-carotene and β-carotene, selenium and zinc, and zinc and copper (all *P* < 0.001). An *r*-value of ≥0.5 was found between iron and copper, β-carotene and α-carotene, selenium and iron, vitamin A and β-carotene, selenium and copper, retinol and vitamin A, vitamin c and β-carotene, vitamin E and copper, copper and vitamin A, and vitamin c and β-cryptoxanthin (all *P* < 0.001), as shown in Figure 2.

### Associations Between 11 Antioxidant Micronutrients and Total CVD

The 11 antioxidant micronutrients were divided into quartiles, and the reference category was considered to be the lowest quartile. The results from the multivariable logistic regression models adjusted for the covariates to assess the prevalence rates of total CVD associated with antioxidant micronutrients are shown in Table 2. Using model 1, 11 antioxidant micronutrients were found to have negative association with total CVD when comparing the second, third, and fourth quartiles with the reference quartile, respectively (all *P* for trend <0.05). Further analysis known risk factors were used as covariates to reduce false positives induced by multiple corrections both in model 2 and 3. The second, third, and fourth quartiles of 11 antioxidant micronutrients were independently associated with the decreased prevalence of total CVD compared with the lowest reference quartile (all *P* for trend < 0.05).

**Table 2:** Adjusted regression coefficients with 95% confidence intervals (95% CIs) in a multiple regression analysis for total CVD model and 11 dietary antioxidant micronutrients in adults from the United States, collected from the National Health, and Nutritional Examination Survey (NHANES) database, 2003–2018.

**Micronutrients**	**Q1**	**Q2**	**Q3**	**Q4**	***P*** **for trend**
	**OR**	**OR (95% CI)**	**OR (95% CI)**	**OR (95% CI)**	
**Vitamin E (mg)**					
Model 1	1	0.83 (0.76–0.91)	0.72 (0.65–0.79)	0.70 (0.64–0.77)	<0.001
Model 2	1	0.86 (0.79–0.94)	0.76 (0.69–0.83)	0.75 (0.68–0.82)	<0.001
Model 3	1	0.85 (0.77–0.93)	0.75 (0.68–0.83)	0.74 (0.67–0.82)	<0.001
**Retinol (μg)**					
Model 1	1	0.89 (0.81–0.98)	0.87 (0.80–0.96)	0.81 (0.74–0.90)	<0.001
Model 2	1	0.91 (0.83–1.00)	0.89 (0.81–0.98)	0.82 (0.74–0.90)	0.001
Model 3	1	0.93 (0.85–1.03)	0.89 (0.81–0.99)	0.85 (0.77–0.94)	0.015
**Vitamin A (μg)**					
Model 1	1	0.87 (0.79–0.95)	0.80 (0.72–0.88)	0.68 (0.62–0.75)	<0.001
Model 2	1	0.89 (0.81–0.98)	0.83 (0.75–0.91)	0.71 (0.64–0.78)	<0.001
Model 3	1	0.89 (0.81–0.99)	0.85 (0.76–0.94)	0.75 (0.68–0.83)	<0.001
**α-carotene (μg)**					
Model 1	1	0.77 (0.69–0.84)	0.76 (0.69–0.83)	0.65 (0.59–0.72)	<0.001
Model 2	1	0.81 (0.73–0.89)	0.82 (0.75–0.91)	0.72 (0.65–0.80)	<0.001
Model 3	1	0.81 (0.73–0.89)	0.81 (0.73–0.89)	0.74 (0.67–0.82)	<0.001
**β-carotene (μg)**					
Model 1	1	0.83 (0.75–0.91)	0.72 (0.65–0.79)	0.65 (0.59–0.71)	<0.001
Model 2	1	0.88 (0.80–0.97)	0.78 (0.71–0.86)	0.71 (0.64–0.78)	<0.001
Model 3	1	0.86 (0.78–0.95)	0.77 (0.70–0.85)	0.75 (0.68–0.83)	<0.001
**β-cryptoxanthin (μg)**					
Model 1	1	0.88 (0.80–0.96)	0.79 (0.72–0.87)	0.77 (0.70–0.85)	<0.001
Model 2	1	0.91 (0.83–1.01)	0.84 (0.76–0.92)	0.83 (0.76–0.92)	<0.001
Model 3	1	0.92 (0.83–1.01)	0.85 (0.77–0.94)	0.90 (0.81–0.99)	0.012
**Vitamin C (mg)**					
Model 1	1	0.79 (0.72–0.87)	0.70 (0.63–0.76)	0.66 (0.60–0.73)	<0.001
Model 2	1	0.84 (0.76–0.92)	0.75 (0.68–0.82)	0.72 (0.66–0.80)	<0.001
Model 3	1	0.84 (0.76–0.93)	0.78 (0.71–0.86)	0.81 (0.73–0.90)	<0.001
**Iron (mg)**					
Model 1	1	0.82 (0.75–0.90)	0.75 (0.68–0.82)	0.69 (0.63–0.77)	<0.001
Model 2	1	0.85 (0.77–0.93)	0.78 (0.71–0.86)	0.73 (0.66–0.80)	<0.001
Model 3	1	0.86 (0.78–0.95)	0.79 (0.72–0.87)	0.74 (0.66–0.82)	<0.001
**Zinc (mg)**					
Model 1	1	0.83 (0.75–0.90)	0.79 (0.72–0.87)	0.73 (0.66–0.81)	<0.001
Model 2	1	0.86 (0.78–0.94)	0.83 (0.75–0.91)	0.76 (0.69–0.85)	<0.001
Model 3	1	0.85 (0.77–0.93)	0.85 (0.77–0.93)	0.76 (0.68–0.85)	<0.001
**Selenium (μg)**					
Model 1	1	0.84 (0.77–0.91)	0.77 (0.70–0.84)	0.65 (0.59–0.72)	<0.001
Model 2	1	0.86 (0.79–0.94)	0.80 (0.73–0.88)	0.70 (0.63–0.78)	<0.001
Model 3	1	0.84 (0.76–0.92)	0.77 (0.70–0.85)	0.67 (0.60–0.75)	<0.001
**Copper (mg)**					
Model 1	1	0.77 (0.71–0.85)	0.72 (0.65–0.79)	0.57 (0.52–0.63)	<0.001
Model 2	1	0.81 (0.74–0.89)	0.78 (0.71–0.85)	0.63 (0.57–0.70)	<0.001
Model 3	1	0.82 (0.74–0.90)	0.79 (0.72–0.87)	0.67 (0.60–0.75)	<0.001

*CVD, cardiovascular disease; OR, Odd ratio; CI, confidence interval; O, quartile*.

*Multivariable logistic regression was conducted, and ORs were calculated while comparing the second, third, and fourth quartiles of each chemical with reference to the first exposure quartile*.

*Model 1 was adjusted as age and sex*.

*Model 2 was adjusted as model 1 plus race, education levels and poverty*.

*Model 3 was adjusted as model 2 plus smoking, drinking, BMI, total cholesterol, dietary supplement use, diabetes and hypertension*.

In model 3, when comparing the fourth quartiles of each antioxidant micronutrients with the reference quartile, vitamin E (OR, 0.74; 95% CI, 0.67–0.82); retinol (OR,0.85; 95% CI, 0.77–0.94); vitamin A (OR, 0.75; 95% CI, 0.68–0.83), α-carotene (OR, 0.74; 95% CI, 0.67–0.82), β-carotene (OR, 0.75; 95% CI, 0.68–0.83), and β-cryptoxanthin (OR, 0.90; 95% CI, 0.81–0.99), vitamin C (OR, 0.81; 95% CI, 0.73–0.90), iron (OR, 0.74; 95% CI, 0.66–0.82), zinc (OR, 0.76; 95% CI, 0.68–0.85), selenium (OR, 0.67; 95% CI, 0.60–0.75) and copper (OR, 0.67; 95% CI, 0.60–0.75) had a lower odds ratio, respectively. The results showed significant protective correlation existed between 11 antioxidant micronutrients and total CVD.

### WQS Regression Analysis of Negative Relationships Between the 11 Antioxidant Micronutrients in Combination and Total and Specific CVDs

The negative relationships between the combined antioxidant micronutrients and prevalence rates of total and specific CVDs were analyzed using WQS regression analysis. The combined index for the 11 antioxidant micronutrients was independently correlated with total CVD (adjusted OR, 0.79; 95% CI, 0.74–0.84; *P* < 0.001), CHF (adjusted OR, 0.82; 95% CI, 0.73–0.91; *P* < 0.001), CHD (adjusted OR, 0.87; 95% CI, 0.79–0.96; *P* = 0.005), angina (adjusted OR, 0.89; 95% CI, 0.79–0.99, *P* = 0.037), heart attack (adjusted OR, 0.86; 95% CI, 0.79–0.94, *P* = 0.001), and stroke (adjusted OR, 0.73; 95% CI, 0.66–0.80, *P* < 0.001), as shown in Table 3. WQS constrains exposure–outcome associations to a negative direction.

**Table 3:** WQS regression model to assess the protective association of the mixture of 11 antioxidant micronutrients with individual CVDs and total CVD risk in adults from (NHANES) database, 2003–2018.

**Subgroup**	**OR**	**95% CI**	***P***
CVD	0.79	(0.74–0.84)	<0.001
Congestive heart failure	0.82	(0.73–0.91)	<0.001
Coronary heart disease	0.87	(0.79–0.96)	0.005
Angina	0.89	(0.79–0.99)	0.037
Heart attack	0.86	(0.79–0.94)	0.001
Stroke	0.73	(0.66–0.80)	<0.001

*CVD, cardiovascular disease; WQS, weighted quantile sum; OR, odds ratio; CI, credibility interval*.

*WQS regression model was adjusted as age, sex, race, education levels, poverty, smoking, drinking, BMI, total cholesterol, dietary supplement use, diabetes and hypertension*.

Selenium was found to have the greatest contribution to the combined effect of the micronutrients (weight = 21.60%) in the total CVD model. Selenium, copper, β-carotene, vitamin E, and iron had weights of >0.1 in the total CVD analysis; additionally, weights of >0.1 were also observed for selenium in CHF, angina and stroke; copper in angina, heart attack, and stroke; vitamin E in CHF, CHD, and heart attack; and iron in CHF and stroke. β-cryptoxanthin had the greatest contribution of all antioxidative micronutrients in stroke (weight = 23.80%); the weight of vitamin C did not exceed 0.1 in the total CVD analysis or those of specific CVDs (as shown in Table 4; Supplementary Figure S1).

**Table 4:** WQS regression analysis of 11 antioxidant micronutrients weights of total CVD and specific CVDs.

	**CVD (%)**	**Congestive heart failure (%)**	**Coronary heart disease (%)**	**Angina (%)**	**Heart attack (%)**	**Stroke (%)**
Selenium	21.60	24.80	2.80	33.00	4.64	14.10
Copper	17.10	9.07	2.48	11.40	27.70	25.00
β-carotene	16.30	19.10	7.86	8.14	5.10	9.47
Vitamin E	11.90	11.20	33.00	6.04	10.90	3.82
Rion	10.60	22.50	8.09	1.860	3.01	12.20
Vitamin C	8.62	0.14	5.50	0.29	2.53	4.25
Vitamin A	4.74	4.43	11.90	17.50	18.20	0.54
α-carotene	4.49	6.60	13.60	12.80	2.61	6.78
Retinol	2.24	0.61	2.87	6.33	18.80	0.04
Zinc	1.46	0.96	10.40	15.70	5.83	0.03
β-cryptoxanthin	0.94	0.51	1.49	00.11	0.71	23.80

*CVD, cardiovascular disease; WQS, weighted quantile sum*.

### Multiple Logistic Regression Analysis for Selenium, Copper, β-Carotene, Vitamin E, and Iron and Specific CVDs

The weight of selenium, copper, β-carotene, vitamin E, and iron were exceeded 0.1, which were significant contribution in CVDs, and the relationship between the 5 antioxidants and specific CVDs was further assessed using multiple logistic regression. After adjustment using model 3, compare with the lowest quartile, as the maximum weight in total CVD model the highest selenium level remained significantly negatively associated with CHF (adjusted OR, 0.70; 95% CI, 0.58–0.84; *P* = 0.001), angina (adjusted OR, 0.70; 95% CI, 0.57–0.85; *P* = 0.002), heart attack (adjusted OR, 0.71; 95% CI, 0.61–0.84, *P* < 0.001), and stroke (adjusted OR, 0.66; 95% CI, 0.55–0.78; *P* < 0.001), respectively (Supplementary Table S2). In addition, copper in the highest quartile remained significant protectively associated with CHF (adjusted OR, 0.73; 95% CI, 0.61–0.87; *P* = 0.002), angina (adjusted OR, 0.74; 95% CI, 0.61–0.89; *P* = 0.017), heart attack (adjusted OR, 0.70; 95% CI, 0.58–0.84; *P* = 0.001) and stroke (adjusted OR, 0.70; 95% CI, 0.58–0.84; *P* = 0.001), respectively (Supplementary Table S3). In the adjusted model, a significant association between fourth quartile of β-carotene and vitamin E and decreased 5 specific CVDs prevalence, respectively (all *P* for trend <0.05) (Supplementary Tables S4, S5); Besides, iron in the second, third, and fourth quartiles decreased risk of CHF, heart attack, and stroke compared to those in the lowest (all *P* for trend <0.05) (Supplementary Table S6).

### Dose Response Relationship Between 11 Antioxidant Micronutrients and the Prevalence Rates of Total CVD and Specific CVDs

**Figure 3:**
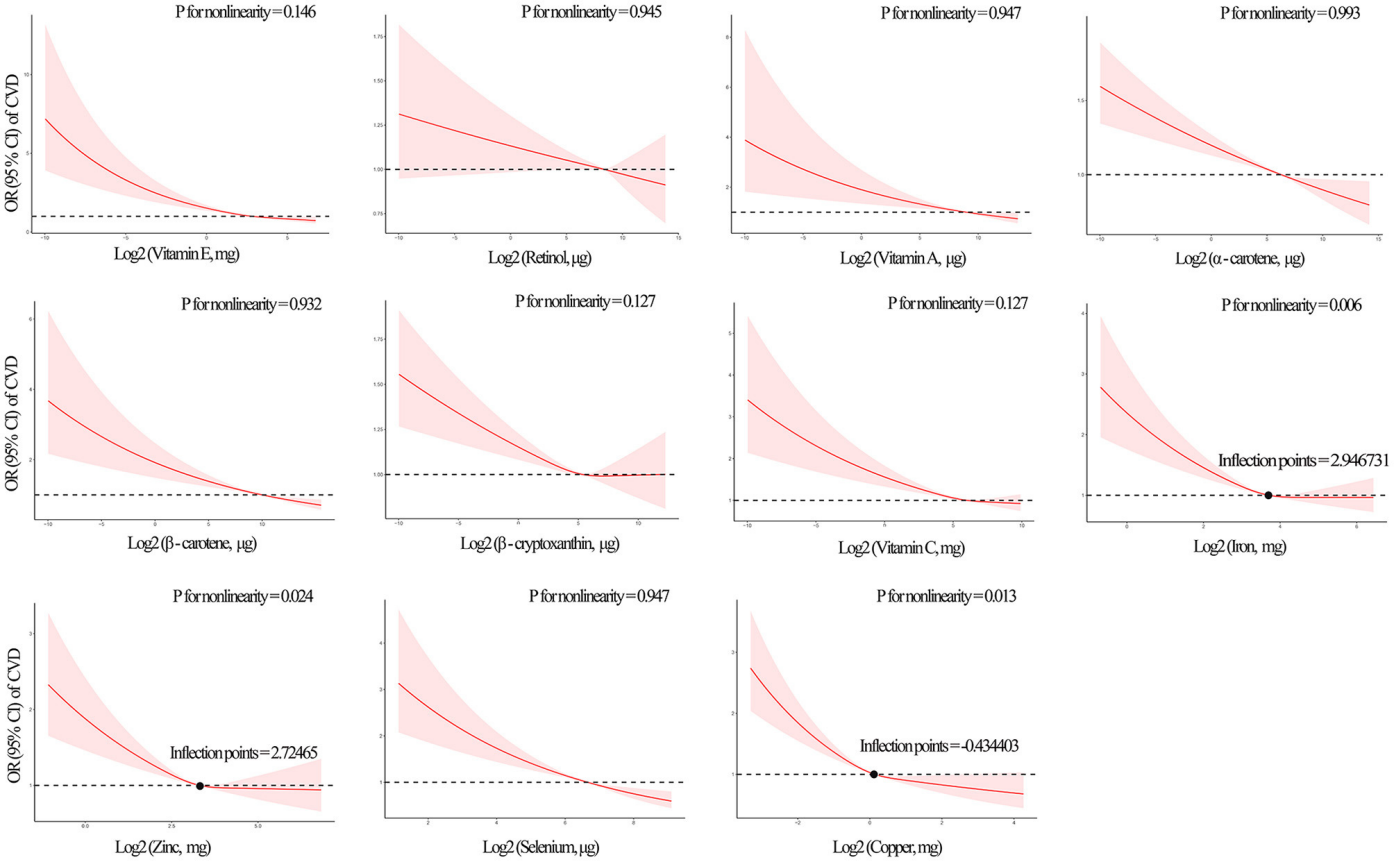
Restricted cubic spline (RCS) analysis with a multivariate -adjusted association associations between dietary 11 antioxidant micronutrients and the prevalence of total CVD. Eleven specific antioxidant micronutrients index are modeled as restricted cubic splines with knots at the 10th, 50th, and 90th percentiles shown the non-linear association. Non-linearly related inflection points of iron, zinc and copper were annotated. The solid line is the adjusted HR; 10th percentile is used as the reference (HR = 1). The shaded area is the 95% CI of the HR. Iron, zinc and copper shown non-linearity association with total CVD model (*P* for non-linearity < 0.05).

The median intake level of dietary iron, zinc and copper were 13.2, 9.9, and 1.1 mg, respectively (Supplementary Table S1). RCS and multivariate logistic regression analyses were used to flexibly model and visualize the U-shaped relationships of iron, zinc and copper with the prevalence of CVD. Iron (*P* for non-linearity = 0.006), zinc (*P* for non-linearity = 0.024) and copper (*P* for non-linearity = 0.013) all had a non-linear and negative correlation with total CVD, and total CVD events increased rapidly for iron, zinc and copper below those levels (Figure 3). Models predicted that the concurrent decrease in risk of CVD with decreased concentrations of iron, zinc and copper flattened out at levels of 7.71, 6.61, and 0.74 mg, respectively (Table 5). All eight other micronutrients had linear, negative associations (all *P* for non-linearity >0.05) (Figure 3).

**Table 5:** Threshold effect analysis of iron, zinc and copper on the prevalence of total CVD risk using piecewise binary logistic regression models.

	**Inflection point**	**group**	**Mean difference (95% CI)**	***P-*** **value**	***P*** **for log likelihood ratio test**
Iron (mg)	7.71	≤7.71	0.83 (0.71–0.97)	0.018	0.221
		>7.71	0.90 (0.84–0.97)	0.007	
Zinc (mg)	6.61	≤6.61	0.75 (0.66–0.86)	<0.001	0.019
		>6.61	0.90 (0.83–0.98)	0.012	
Copper (mg)	0.74	≤0.74	0.71 (0.61–0.83)	<0.001	0.025
		>0.74	0.86 (0.80–0.94)	<0.001	

*OR, Odd ratio; CI, confidence interval*.

*Iron, zinc and copper were log 2 transformed for fitting the piecewise binary logistic regression model*.

*Analyses was adjusted for age, sex, race, education levels, poverty, smoking, drinking, BMI, total cholesterol, dietary supplement use, diabetes and hypertension*.

The relationships between each of the 11 microelements and specific CVDs (CHD, CHF, heart attack, angina, and stroke) had negative non-linear and linear outcomes as shown in Supplementary Figures S2–S6. Among these, the association of β-cryptoxanthin with CHF, and iron, zinc, and copper with heart attack (non-linearity, *P* < 0.05) were all negative and non-linear. Linear, negative associations were found between each of the 11 microelements and stroke (all *P* for non-linearity >0.05).

## Discussion

Our analysis of 11 dietary antioxidant micronutrients in 39,757 US adults from a prospective, nationally representative U.S. cohort demonstrated that, after adjustment for common confounding variables, all individual 11 micronutrients were independently negatively associated with total CVD. In addition, the WQS index model showed a negative correlation between all the antioxidative micronutrients in combination and total CVD, with the greatest influence on this relationship from selenium. Negative associations were also observed between selenium and several specific CVDs (CHF, CHD, angina, heart attack, stroke). Non-linearity regression indicated a U-shaped correlation between iron, zinc and copper and total CVD, with inflection points at 7.71, 6.61, and 0.74 mg/day, respectively.

Oxidative stress exceeds the buffering capacity of the antioxidant defense systems ultimately resulting in cardiovascular dysfunction (22, 38). Laboratory data shown exogenous antioxidants, as a part of a diet are able to protect tissues from ROS and reactive RNS-induced injury (3, 39), and thus protect cells and organ systems against free radical damage. For instance, Vitamins A, C, and E and carotenoids direct the neutralization of free radicals; break the chain reaction of lipid peroxidation (40), lowering total cholesterol and low-density lipoprotein and CVD risks (41); copper, zinc, iron and selenium are required for the activity of superoxide dismutase (SOD), catalase and glutathione peroxidase (GPx) diminish excessive oxidative stress (42, 43). Current our partial outcomes were consistent with previous population large meta-analyses and a few randomized controlled clinical trials, which have shown that single magnesium, copper and zinc (42, 44), selenium, carotenoids (17), vitamin C (44), and vitamin E (45, 46), are all associated with reduced prevalence of CVDs, and CVD-related death.

Despite the theoretical that these antioxidants must exert beneficial effects against oxidative stress, other prospective population trials have yielded inconsistent results on their ability to prevent CVD (47). Dosage was one of the main reasons proposed for this inconsistency. Intake of β -carotene at 15–50 mg/day had no beneficial effects on CVD outcomes in adults in a meta-analysis consisting of 15 clinical trials (40, 48). It's also notable that immoderate removal of ROS or RNS and their derived products by antioxidant supplementation may upset cell signaling pathways and might be increase the risk of chronic disease (49). Research on the effect of excess antioxidant supplementation on CVDs reported that selenium in the diet at >400 μg/day induced selenosis and heart hazards (50), and 4 years of supplementation with 20–30 mg/day β-carotene was associated with increased risk of CVD (51). Moreover, an upper limit for vitamin A (retinol) at 3,000 μg/day in adults is extrapolated from a small number of case reports (15, 52).

It is difficult to obtain such high levels of trace elements from conventional foods; thus, micronutrients obtained in the diet rather than through supplements could be considered safer (45). Previous studies highlighted those whole grains, nuts, seeds, fruits and vegetables are rich in essential antioxidative micronutrients, which as role in the primary prevention of patients with CVD (9), and those who are at risk (11). Higher vegetable and nut intakes have been associated with a lower risk of stroke: an increase of 1 serving per day of green leafy vegetables intake yielded a relative risk (RR) of 0.79 (95% CI, 0.62–0.99) (42, 53); similar effects were demonstrated for myocardial infarction (0.5 servings per week vs. once a week, RR = 0.49) (39, 54). To date, antioxidants with safer dietary profile [vitamin A at 3,000 μg/day or 7,500 μg/week; α-carotenoids at up to 20 mg/day for lutein and 75 mg/day for lycopene; β-carotene at 2–4 mg/day (14, 15, 48); selenium at 55 μg/day (19) or 200 μg/day for 12 weeks (20)]; vitamin C at 500–700 mg/day (13, 14) have been confirmed, and reductions in CVDs and markers of cardiometabolic risk in adults have been observed with their use. In our study, the antioxidant micronutrients came from food rather than supplementation. Supplementary Table S1 shown the daily 95th percentile of antioxidants (vitamin E, 21.8 mg; retinol, 1,201.0 μg; vitamin A, 1,414.0 μg; α-carotene, 1,785.1 μg; β-carotene, 7,816.1 μg; β-cryptoxanthin, 397.5 μg; vitamin C, 233.9 mg; iron, 28.7 mg; zinc, 22 mg; selenium, 207.8 μg; and copper, 2.3 mg) within safe limits had a cardiovascular protective effect after adjustment for confounding factors in our multiple logistic regression. Our non-linear regression indicated that intakes of iron, zinc and copper of 7.71, 6.61, and 0.74 mg/day may decrease total CVD risk, and also provides reference dose levels for future prospective studies.

Another important factor to consider is the antioxidants ability to prevent CVD relies on the internal antioxidant network mechanism of multiple micronutrients. For example, the fat-soluble vitamins A and E have been evaluated for their synergistic effects on GSH homeostasis and antioxidant properties (55). Vitamin C is known to act with vitamin E to regenerate α-tocopherol in membranes and lipoproteins, playing an important role in protein thiol group protection against oxidation (56). The pro-oxidant vs. antioxidant activity of beta-carotene and lycopene has also been found to depend on their interaction with other co-antioxidant molecules such as vitamin C or E in biological membranes (57). In a mineral antioxidant study, vitamins also showing a protective association between zinc metabolic level influence cardiometabolic risk factor (58). Thus, the single-nutrient approach to nutritional epidemiology is far from sufficient to explain the biological effects of antioxidant micronutrients. The field of nutritional health is shifting toward studying the effects of exposure to combinations of nutrients, and the contribution of its individual components on health outcomes. In this study, WQS regression index showed that the combination of 11 antioxidant micronutrients were negatively associated with CVD. The current study focuses on overall and accurate dietary antioxidant micronutrients data collection, preliminary exploring correlations between individual or combined micronutrients and CVDs. Also found that selenium has the greatest influence on the association between all 11 micronutrients in combination and total CVD.

Selenium is considered a cornerstone of the body's antioxidant defense mechanism; this is because it is incorporated in various enzymes with antioxidant and anti-inflammatory functions. Thirteen prospective cohort (59) and observational studies (60) found a moderate inverse relationship between plasma/serum selenium and CHD (61). A U-shaped relationship between serum selenium levels and cardiovascular mortality may account for conflicting observational reports from NHANES study (62). RCS regression in our study showed linear, negative associations of selenium with both total CVD and specific CVDs (stroke, CHD, CHF, heart attack and angina). Our study investigated the 95th percentiles of dietary selenium intake, which was 207.8 μg/day; large-scale, randomized controlled trials have investigated supplementation with a selenium antioxidant cocktail with a daily dose between 75 and 200 μg or selenium supplementation at 100 μg/day over 12 consecutive months did not show any major benefits of selenium cardiovascular endpoints or left ventricular systolic dysfunction, likely due to underdosage (20).

Our study has some strengths. We selected WQS over alternative approaches in our study because it accounts for exposure-outcome correlations, as well as correlations between exposures, highlighting the contribution of individual components of the combination (63). WQS regression also conserves statistical power and prevents unstable regression coefficients, which might otherwise occur if the highly-correlated antioxidant micronutrients were included simultaneously in traditional regression models. A diet-based, rather than supplement-based, approach to nutritional interventions in CVD had proven to be an effective strategy resulting in strong and tangible results. Micronutrients in the diet have synergistic effects, and incorporating this synergy in the development of dietary recommendations is therefore likely to provide the maximum obtainable benefit obtainable from nutrition.

Our study also has limitations to consider. First, WQS regression requires a directional homogeneity assumption, which assumes that either all exposures have adjusted associations with the outcome that are all in a positive or a negative direction (or can be coded a priori to meet this assumption), or associations are null. However, the antioxidant micronutrients in the study were all shown to be individually negatively associated with CVD, satisfying this assumption. Second, WQS regression also assumes the individual micronutrients have linear and additive effects. Little is known about the benefits of these assumptions and whether they have adverse impacts on studies of epidemiologic data, in which such assumptions can never be met precisely (27). However, this method provides a parsimonious, parametric inference of the effect of combinations of factors, and was therefore a highly appropriate method to use in this study. Third, although the NHANES use of 24-h dietary recall was the preferred choice of reference method. Reliance on memory with twice recall is a well-documented limitation might induce bias for micronutrients calculation. Comprehensive and additional dietary data in NHANES have much more detailed information would be conducive to minimize possibility of self-reporting bias (28). Fourth, although the potential role of the 11 antioxidant micronutrients in the development and progression of CVDs has been investigated, the accuracy of self-reported outcomes in NHANES is not well-characterized. The chemical forms of antioxidant micronutrients (as organic and inorganic compounds) and sustained and dynamic intakes should also be considered. Building on our study, which has demonstrated important synergistic effects of antioxidant micronutrients, randomized trials with standardized antioxidants and protocols would help to improve our understanding of the effects of different food combinations on cardiovascular outcomes.

## Conclusion

Our findings suggested that higher levels of antioxidative micronutrients in combination are associated with a decreased total CVD risk, and that selenium has the greatest contribution to this effect. Significant negative linear and non-linear correlations exist between the 11 antioxidative micronutrients and total CVD or specific CVDs. Future research is needed to better understand the interactions and complexities of multiple micronutrients and their combined effects on cardiovascular health, and to assess and identify optimal intake levels to reduce CVD in at-risk populations.

## Data Availability Statement

Publicly available datasets were analyzed in this study. This data can be found here: https://www.cdc.gov/nchs/nhanes/index.htm.

## Ethics Statement

Ethical review and approval was not required for the study on human participants in accordance with the local legislation and institutional requirements. The patients/participants provided their written informed consent to participate in this study.

## Author Contributions

TY and XZ designed research, drafted the manuscript, and performed statistical analysis. DX and HL extracted the data and conducted analyses. XLu, YT, and MS took charge of software operation. WY and YZ reviewed the manuscript. XLi and HZ conceptualized the study. All authors reviewed, edited, and finalized the final version of the manuscript.

## Funding

This work was supported by Key Disciplines of The First Affiliated Hospital of Nanjing Medical University.

## Conflict of Interest

The authors declare that the research was conducted in the absence of any commercial or financial relationships that could be construed as a potential conflict of interest.

## Publisher's Note

All claims expressed in this article are solely those of the authors and do not necessarily represent those of their affiliated organizations, or those of the publisher, the editors and the reviewers. Any product that may be evaluated in this article, or claim that may be made by its manufacturer, is not guaranteed or endorsed by the publisher.

## Supplementary Material

Supplementary Figure S1 Weights from the weighted quantile sum (WQS) regression index for dietary intake of 11 antioxidant micronutrients and the prevalence of total cardiovascular disease (CVD). A protective (negatively-associated) model is shown, adjusted for age, sex, race, education level, smoker status, poverty, body mass index (BMI), and diabetes mellitus status.

Supplementary Figures S2–S6 Restricted cubic spline (RCS) analysis with a multivariate -adjusted association associations between dietary eleven antioxidant micronutrients and the prevalence of specific CVDs (CHD, CHF, heart attack, angina and stroke). Eleven antioxidant micronutrients index are modeled as restricted cubic splines with knots at the 10th, 50th, and 90th percentiles shown the non-linear association. The solid line is the adjusted HR; 10th percentile is used as the reference (HR = 1). The shaded area is the 95% CI of the HR. The β–cryptoxanthin shown non-linearity association with CHF model (*P* for non-linearity <0.05), and other 10 micronutrients shown linearity correlation with CHF (*P* for non-linearity > 0.05). The iron, zinc and copper shown non-linearity association with heart attack (*P* for non-linearity <0.05), others shown linearity correlation relationship with heart attack (*P* for non-linearity >0.05). All 11 specific antioxidant micronutrients shown linearity association with CHD, angina, and stroke (*P* for non-linearity > 0.05).

Supplementary Table 1 Concentrations and distribution of the eleven antioxidant micronutrients.

Supplementary Table 2 Multiple logistic regression model to assess the association between selenium and specific CVD risk.

Supplementary Table 3 Logistic regression model to assess the association between copper and specific CVD risk.

Supplementary Table 4 Logistic regression model to assess the association between β-carotene and specific CVD risk.

Supplementary Table 5 Logistic regression model to assess the association between vitamin E and specific CVD risk.

Supplementary Table 6 Logistic regression model to assess the association between iron and specific CVD risk.
