# Supplementary figures and images for: The Association Between Dietary Antioxidant Micronutrients and Cardiovascular Disease in Adults in the United States: A Cross-Sectional Study
Source: Front Nutr. 2022 Jan 12;8:799095. doi: 10.3389/fnut.2021.799095 (PMC8791653; doi:10.3389/fnut.2021.799095)

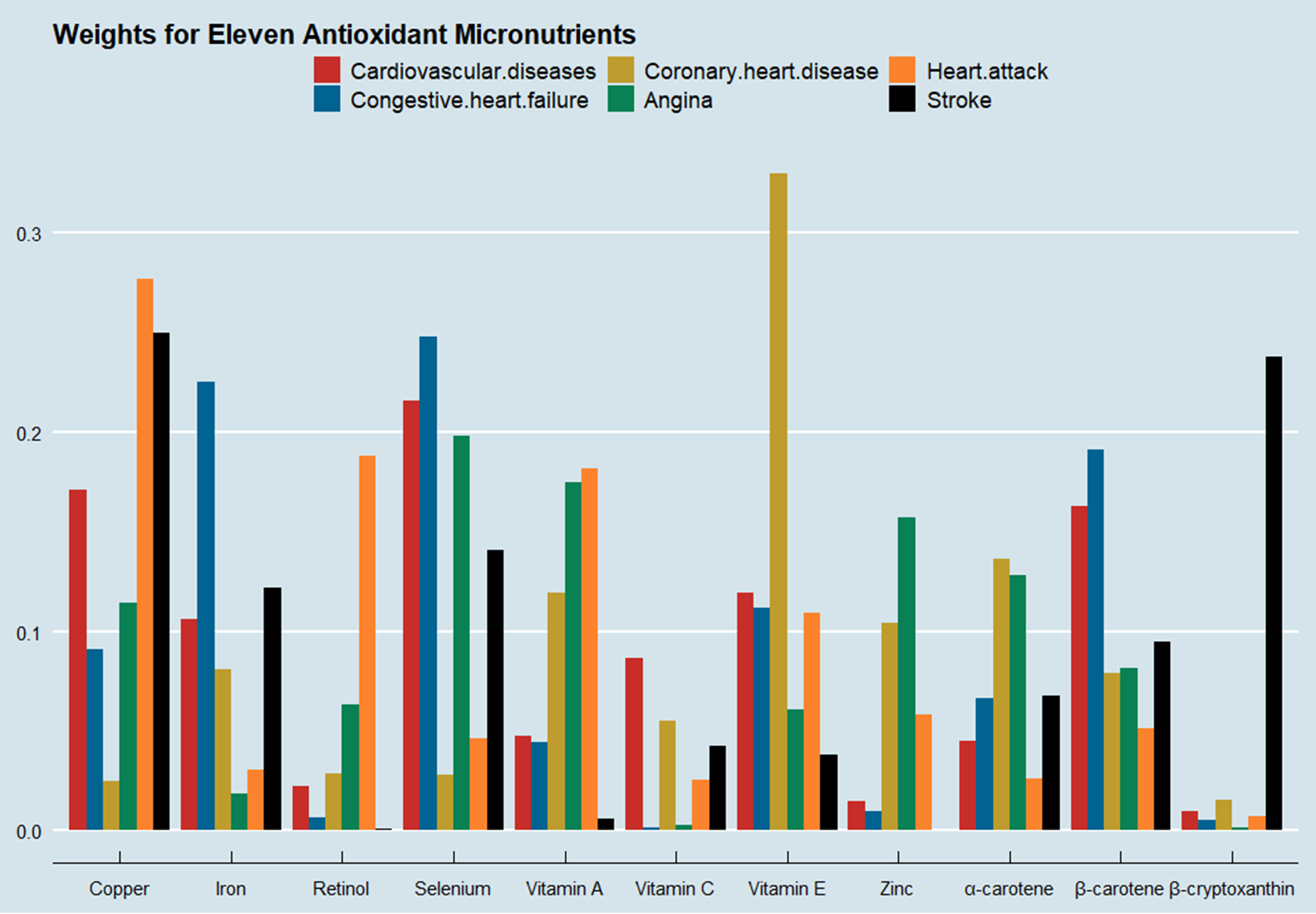

Supplement: Supplementary Figure S1 — Weights from the weighted quantile sum (WQS) regression index for dietary intake of 11 antioxidant micronutrients and the prevalence of total cardiovascular disease (CVD). A protective (negatively-associated) model is shown, adjusted for age, sex, race, education level, smoker status, poverty, body mass index (BMI), and diabetes mellitus status. [file Image_1.TIF]

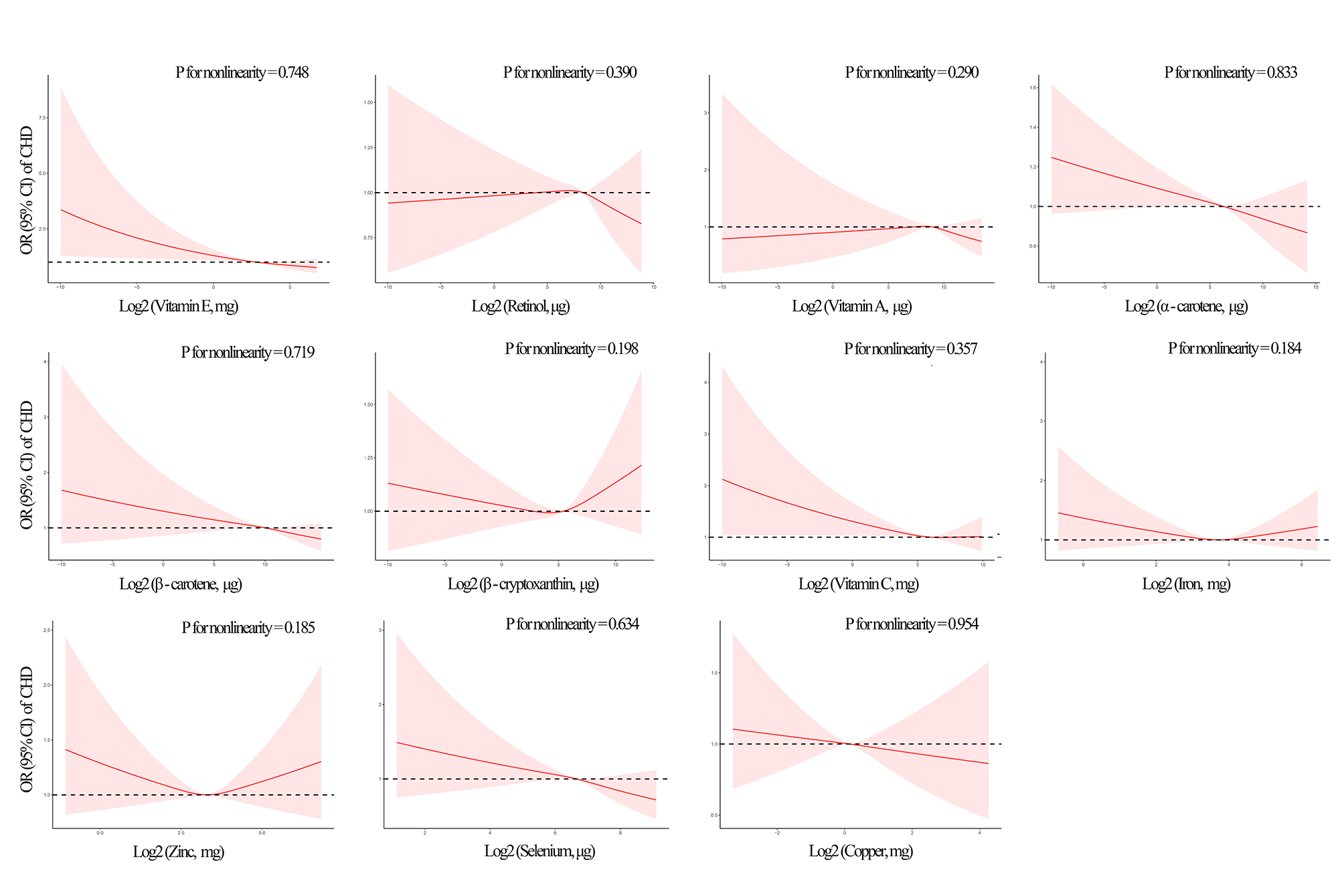

Supplement: Supplementary file 2 [file Image_2.JPG]

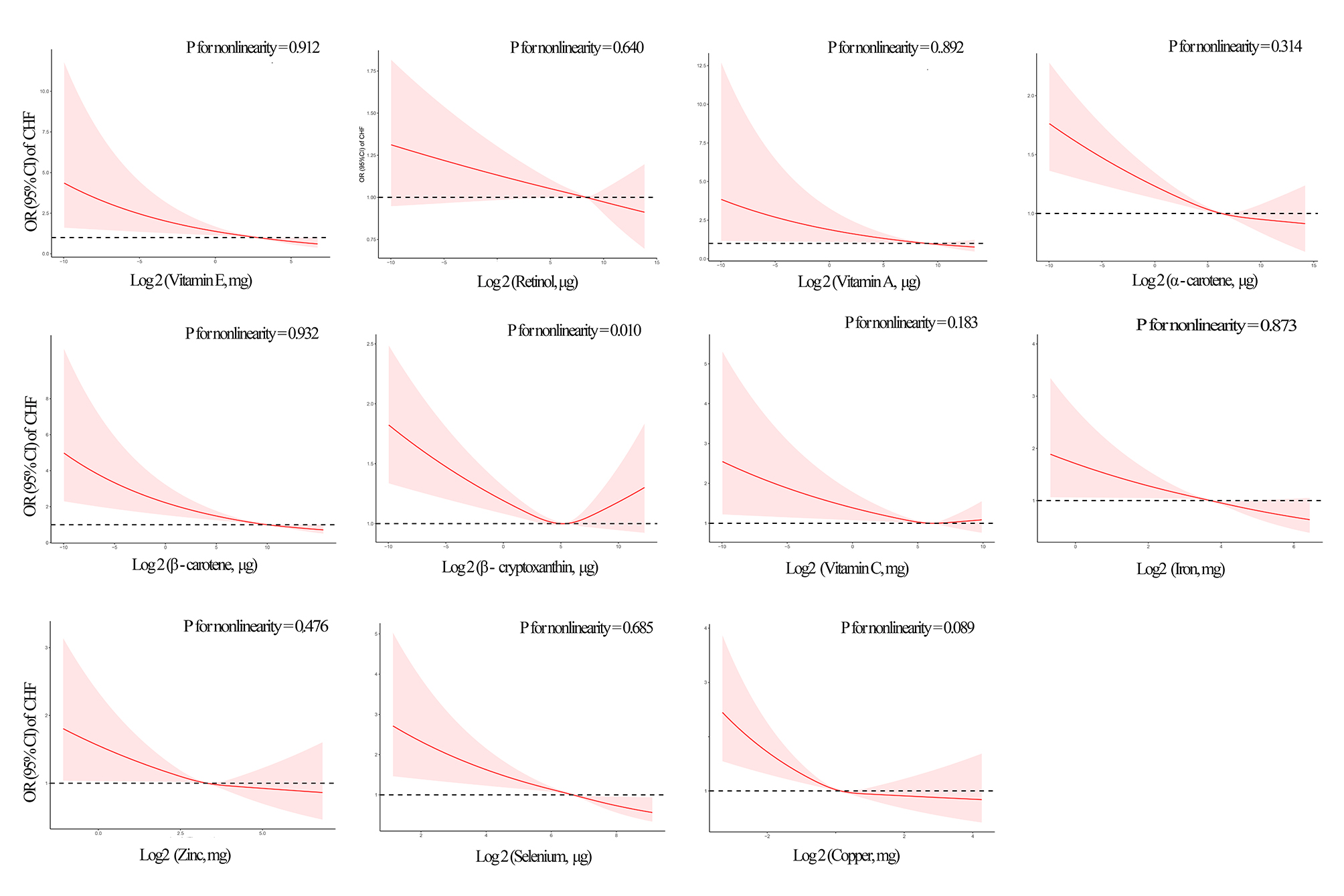

Supplement: Supplementary file 3 [file Image_3.JPG]

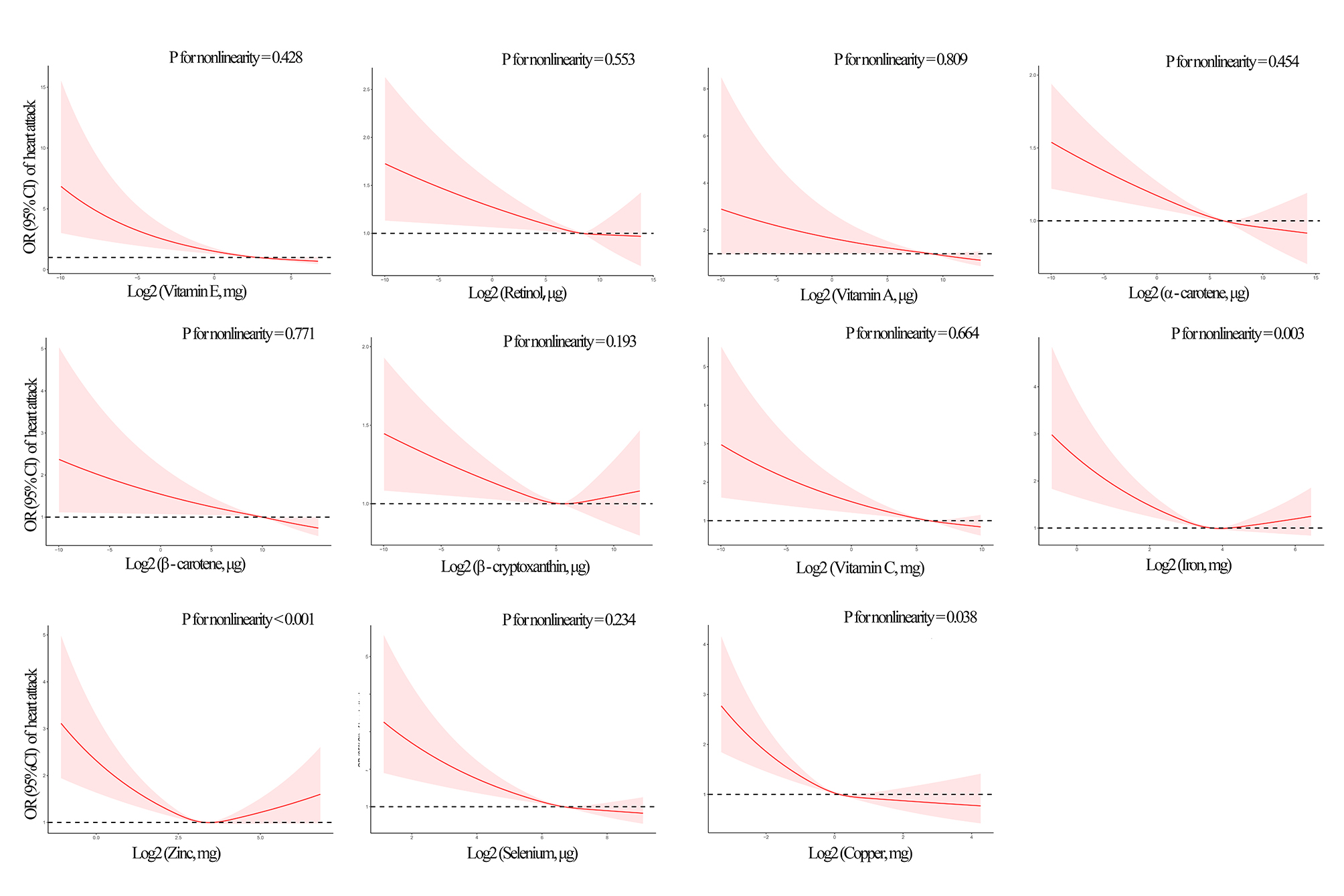

Supplement: Supplementary file 4 [file Image_4.JPG]

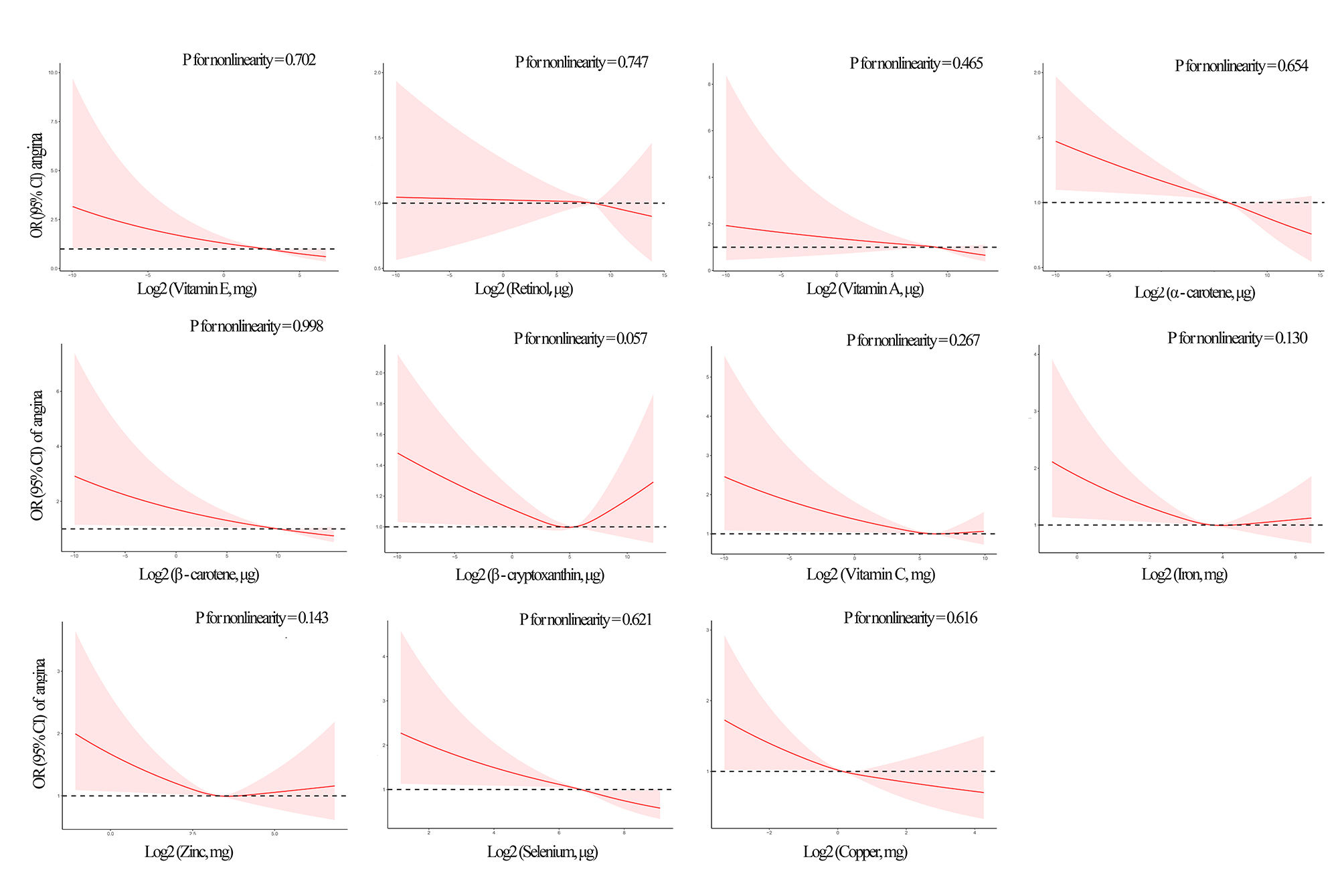

Supplement: Supplementary file 5 [file Image_5.JPG]

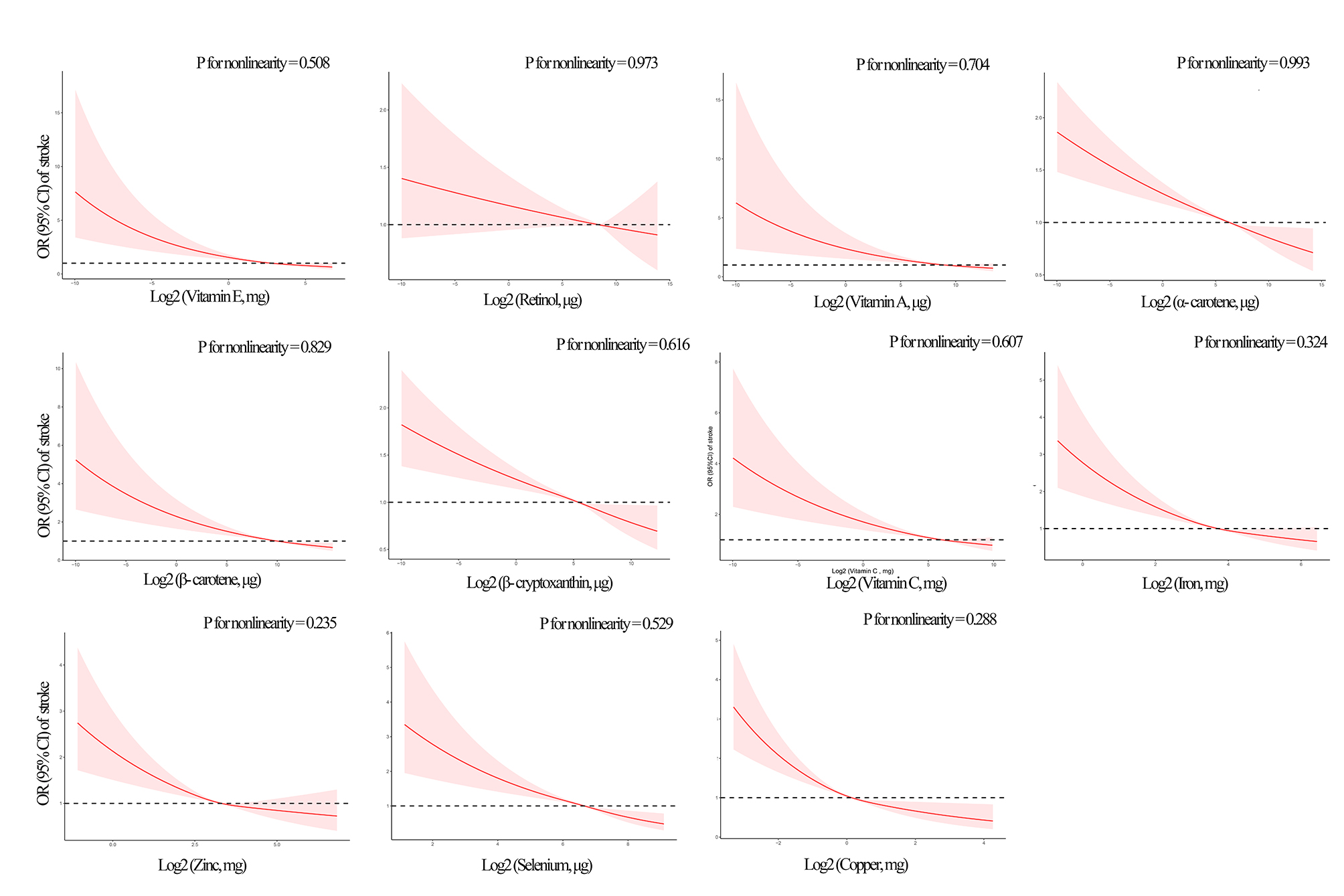

Supplement: Supplementary Figures S2–S6 — Restricted cubic spline (RCS) analysis with a multivariate -adjusted association associations between dietary eleven antioxidant micronutrients and the prevalence of specific CVDs (CHD, CHF, heart attack, angina and stroke). Eleven antioxidant micronutrients index are modeled as restricted cubic splines with knots at the 10th, 50th, and 90th percentiles shown the non-linear association. The solid line is the adjusted HR; 10th percentile is used as the reference (HR = 1). The shaded area is the 95% CI of the HR. The β–cryptoxanthin shown non-linearity association with CHF model (P for non-linearity <0.05), and other 10 micronutrients shown linearity correlation with CHF (P for non-linearity > 0.05). The iron, zinc and copper shown non-linearity association with heart attack (P for non-linearity <0.05), others shown linearity correlation relationship with heart attack (P for non-linearity >0.05). All 11 specific antioxidant micronutrients shown linearity association with CHD, angina, and stroke (P for non-linearity > 0.05). [file Image_6.JPG]
