# Supplementary material for: The Association Between Dietary Antioxidant Micronutrients and Cardiovascular Disease in Adults in the United States: A Cross-Sectional Study
Source: Front Nutr. 2022 Jan 12;8:799095. doi: 10.3389/fnut.2021.799095 (PMC8791653; doi:10.3389/fnut.2021.799095)
Supplement: Supplementary Table 1 — Concentrations and distribution of the eleven antioxidant micronutrients. [file Table_1.DOCX]

**Supplement Table 1.** Concentrations and distribution of the eleven antioxidant micronutrients.

| Micronutrients | N | 5^th^ | 25^th^ | 50^th^ | 75^th^ | 95^th^ |
| --- | --- | --- | --- | --- | --- | --- |
| Vitamin E (mg) | 39757 | 2.0 | 4.3 | 6.9 | 10.8 | 21.8 |
| Retinol (μg) | 39757 | 31.0 | 161.0 | 327.0 | 576.0 | 1201.0 |
| Vitamin A (μg) | 39757 | 119.0 | 304.0 | 500.5 | 772.5 | 1414.0 |
| α-carotene (μg) | 39757 | 1.0 | 23.0 | 78.5 | 422.3 | 1785.1 |
| β-carotene (μg) | 39757 | 114.0 | 415.5 | 1031.0 | 2655.0 | 7816.1 |
| β-cryptoxanthin (μg) | 39757 | 1.5 | 13.5 | 42.5 | 114.0 | 397.5 |
| Vitamin C (mg) | 39757 | 8.2 | 29.7 | 64.0 | 116.9 | 233.9 |
| Iron (mg) | 39757 | 5.7 | 9.6 | 13.2 | 18.0 | 28.7 |
| Zinc (mg) | 39757 | 4.1 | 7.1 | 9.9 | 13.7 | 22.0 |
| Selenium (μg) | 39757 | 43.6 | 73.8 | 101.2 | 135.5 | 207.8 |
| Copper (mg) | 39757 | 0.5 | 0.8 | 1.1 | 1.5 | 2.3 |

Abbreviations: N, number of participants; 5^th^, 5th percentile; 25^th^, 25th percentile; 50^th^, 50th percentile; 75^th^, 75th percentile; 95^th^, 95th percentile.

**Supplementary table 2.** Multiple logistic regression model to assess the association between selenium and specific CVD risk.

| Specific CVD | Q1 | Q2 | Q3 | | Q4 | | *P* for trend | |
| --- | --- | --- | --- | --- | --- | --- | --- | --- |
|  | OR | OR (95% CI) | OR (95% CI) | | OR (95% CI) | |  |  |
| **Congestive heart failure** | | | | | | | | |
| Model 1 | 1 | 0.85(0.73-0.98) | | 0.77(0.66-0.90) | | 0.66(0.55-0.79) | | <0.001 |
| Model 2 | 1 | 0.89(0.77-1.03) | | 0.83(0.71-0.97) | | 0.73(0.61-0.88) | | 0.006 |
| Model 3 | 1 | 0.86(0.74-1.00) | | 0.79(0.67-0.93) | | 0.70(0.58-0.84) | | 0.001 |
| **Coronary heart disease** | | | | | | | | |
| Model 1 | 1 | 0.95(0.83-1.09) | | 0.97(0.84-1.12) | | 0.84(0.71-0.98) | | 0.153 |
| Model 2 | 1 | 0.93(0.81-1.07) | | 0.95(0.82-1.10) | | 0.83(0.71-0.98) | | 0.165 |
| Model 3 | 1 | 0.92(0.79-1.05) | | 0.93(0.80-1.07) | | 0.82(0.69-0.96) | | 0.115 |
| **Angina** | | | | | | | | |
| Model 1 | 1 | 0.87(0.74-1.02) | | 0.82(0.70-0.98) | | 0.72(0.59-0.87) | | 0.007 |
| Model 2 | 1 | 0.86(0.73-1.01) | | 0.81(0.69-0.97) | | 0.72(0.59-0.88) | | 0.008 |
| Model 3 | 1 | 0.84(0.71-0.98) | | 0.79(0.66-0.93) | | 0.70(0.57-0.85) | | 0.002 |
| **Heart attack** | | | | | | | | |
| Model 1 | 1 | 0.83(0.73-0.95) | | 0.80(0.70-0.92) | | 0.68(0.58-0.79) | | <0.001 |
| Model 2 | 1 | 0.85(0.74-0.97) | | 0.84(0.73-0.96) | | 0.73(0.62-0.85) | | 0.001 |
| Model 3 | 1 | 0.83(0.73-0.95) | | 0.81(0.71-0.94) | | 0.71(0.61-0.84) | | <0.001 |
| **Stroke** | | | | | | | | |
| Model 1 | 1 | 0.77(0.67-0.88) | | 0.63(0.54-0.72) | | 0.60(0.51-0.71) | | <0.001 |
| Model 2 | 1 | 0.80(0.70-0.92) | | 0.67(0.58-0.78) | | 0.65(0.55-0.77) | | <0.001 |
| Model 3 | 1 | 0.79(0.69-0.90) | | 0.66(0.57-0.77) | | 0.66(0.55-0.78) | | <0.001 |

Abbreviations: CVD, cardiovascular disease; OR, Odd ratio; CI, confidence interval, O, quartile.

Selenium (μg) was divided to four levels by quartile (Q1 ≤ 73.8; 73.8 < Q2 ≤ 101.2; 101.2 < Q3≤ 135.5; Q4 > 135.5).

Model 1 was adjusted as age and sex;

Model 2 was adjusted as model 1 plus race, education levels and poverty;

Model 3 was adjusted as model 2 plus smoking, drinking, BMI, total cholesterol, dietary supplement use, diabetes and hypertension.

**Supplement Table 3.** Logistic regression model to assess the association between copper and specific CVD risk.

| Specific CVD | Q1 | Q2 | | Q3 | Q4 | | *P* for trend | |
| --- | --- | --- | --- | --- | --- | --- | --- | --- |
|  | OR | OR (95% CI) | | OR (95% CI) | OR (95% CI) | |  |  |
| **Congestive heart failure** | | | | | | | | |
| Model 1 | 1 | 0.75 (0.65, 0.87) | 0.72 (0.61, 0.83) | | | 0.57 (0.48, 0.68) | | <0.001 |
| Model 2 | 1 | 0.81 (0.70, 0.94) | 0.80 (0.69, 0.94) | | | 0.66 (0.56, 0.79) | | <0.001 |
| Model 3 | 1 | 0.81 (0.69, 0.94) | 0.82 (0.70, 0.97) | | | 0.73 (0.61, 0.87) | | 0.002 |
| **Coronary heart disease** | | | | | | | | |
| Model 1 | 1 | 0.91 (0.79, 1.05) | 0.94 (0.82, 1.08) | | | 0.79 (0.68, 0.92) | | 0.021 |
| Model 2 | 1 | 0.89 (0.77, 1.02) | 0.91 (0.79, 1.06) | | | 0.75 (0.64, 0.88) | | 0.005 |
| Model 3 | 1 | 0.90 (0.77, 1.03) | 0.94 (0.81, 1.09) | | | 0.81 (0.69, 0.95) | | 0.060 |
| **Angina** | | | | | | | | |
| Model 1 | 1 | 0.86 (0.73, 1.01) | 0.84 (0.71, 0.99) | | | 0.70 (0.58, 0.84) | | 0.002 |
| Model 2 | 1 | 0.84 (0.72, 0.99) | 0.82 (0.69, 0.97) | | | 0.68 (0.56, 0.82) | | 0.001 |
| Model 3 | 1 | 0.86 (0.72, 1.01) | 0.84 (0.71, 1.00) | | | 0.74 (0.61, 0.89) | | 0.017 |
| **Heart attack** | | | | | | | | |
| Model 1 | 1 | 0.74 (0.65, 0.85) | 0.71 (0.62, 0.81) | | | 0.57 (0.49, 0.66) | | <0.001 |
| Model 2 | 1 | 0.77 (0.67, 0.88) | 0.76 (0.66, 0.87) | | | 0.62 (0.53, 0.72) | | <0.001 |
| Model 3 | 1 | 0.78 (0.68, 0.90) | 0.78 (0.68, 0.90) | | | 0.67 (0.57, 0.78) | | <0.001 |
| **Stroke** | | | | | | | | |
| Model 1 | 1 | 0.69 (0.60, 0.78) | 0.61 (0.53, 0.70) | | | 0.45 (0.38, 0.53) | | <0.001 |
| Model 2 | 1 | 0.74 (0.65, 0.84) | 0.68 (0.59, 0.79) | | | 0.52 (0.44, 0.61) | | <0.001 |
| Model 3 | 1 | 0.75 (0.66, 0.86) | 0.71 (0.61, 0.82) | | | 0.55 (0.47, 0.66) | | <0.001 |

Abbreviations: OR, Odd ratio; CI, confidence interval; Q, quartile.

Copper (μg) was divided to four levels by quartile (Q1 ≤ 0.8; 0.8 < Q2 ≤ 1.1; 1.1 < Q3≤ 1.5; Q4 > 1.5).

Model 1 was adjusted as age and sex;

Model 2 was adjusted as model 1 plus race, education levels and poverty;

Model 3 was adjusted as model 2 plus smoking, drinking, BMI, total cholesterol, dietary supplement use, diabetes and hypertension.

**Supplement Table 4.** Logistic regression model to assess the association between β-carotene and specific CVD risk.

| Specific CVD | Q1 | Q2 | | Q3 | Q4 | | *P* for trend | |
| --- | --- | --- | --- | --- | --- | --- | --- | --- |
|  | OR | OR (95% CI) | | OR (95% CI) | OR (95% CI) | |  |  |
| **Congestive heart failure** | | | | | | | | |
| Model 1 | 1 | 0.84 (0.72, 0.98) | 0.69 (0.59, 0.81) | | | 0.61 (0.52, 0.72) | | <0.001 |
| Model 2 | 1 | 0.91 (0.78, 1.07) | 0.78 (0.66, 0.91) | | | 0.69 (0.58, 0.81) | | <0.001 |
| Model 3 | 1 | 0.90 (0.77, 1.05) | 0.77 (0.65, 0.91) | | | 0.73 (0.62, 0.87) | | 0.001 |
| **Coronary heart disease** | | | | | | | | |
| Model 1 | 1 | 0.99 (0.85, 1.14) | 0.84 (0.72, 0.97) | | | 0.80 (0.69, 0.92) | | 0.003 |
| Model 2 | 1 | 0.98 (0.84, 1.13) | 0.82 (0.71, 0.96) | | | 0.81 (0.70, 0.94) | | 0.004 |
| Model 3 | 1 | 0.96 (0.82, 1.11) | 0.81 (0.70, 0.94) | | | 0.85 (0.73, 0.98) | | 0.018 |
| **Angina** | | | | | | | | |
| Model 1 | 1 | 0.86 (0.72, 1.02) | 0.87 (0.74, 1.03) | | | 0.69 (0.58, 0.82) | | 0.001 |
| Model 2 | 1 | 0.85 (0.71, 1.01) | 0.87 (0.73, 1.03) | | | 0.71 (0.59, 0.84) | | 0.002 |
| Model 3 | 1 | 0.84 (0.70, 1.00) | 0.87 (0.73, 1.03) | | | 0.75 (0.63, 0.90) | | 0.018 |
| **Heart attack** | | | | | | | | |
| Model 1 | 1 | 0.86 (0.74, 0.98) | 0.79 (0.69, 0.90) | | | 0.67 (0.58, 0.77) | | <0.001 |
| Model 2 | 1 | 0.91 (0.79, 1.04) | 0.85 (0.74, 0.98) | | | 0.74 (0.64, 0.86) | | 0.001 |
| Model 3 | 1 | 0.90 (0.78, 1.04) | 0.86 (0.74, 0.99) | | | 0.80 (0.69, 0.92) | | 0.020 |
| **Stroke** | | | | | | | | |
| Model 1 | 1 | 0.75 (0.65, 0.87) | 0.61 (0.53, 0.70) | | | 0.58 (0.50, 0.67) | | <0.001 |
| Model 2 | 1 | 0.82 (0.71, 0.95) | 0.69 (0.59, 0.79) | | | 0.64 (0.55, 0.75) | | <0.001 |
| Model 3 | 1 | 0.81 (0.70, 0.94) | 0.69 (0.59, 0.80) | | | 0.68 (0.58, 0.78) | | <0.001 |

Abbreviations: OR, Odd ratio; CI, confidence interval; Q, quartile.

β-carotene (μg) was divided to four levels by quartile (Q1 ≤ 415.5; 415.5 < Q2 ≤ 1031.0; 1031.0 < Q3≤ 2655.0; Q4 > 2655.0).

Model 1 was adjusted as age and sex;

Model 2 was adjusted as model 1 plus race, education levels and poverty;

Model 3 was adjusted as model 2 plus smoking, drinking, BMI, total cholesterol, dietary supplement use, diabetes and hypertension.

**Supplement Table 5.** Logistic regression model to assess the association between vitamin E and specific CVD risk.

| Specific CVD | Q1 | Q2 | Q3 | | Q4 | | *P* for trend | |
| --- | --- | --- | --- | --- | --- | --- | --- | --- |
|  | OR | OR (95% CI) | OR (95% CI) | | OR (95% CI) | |  |  |
| **Congestive heart failure** | | | | | | | | |
| Model 1 | 1 | 0.89 (0.77, 1.03) | | 0.83 (0.71, 0.96) | | 0.66 (0.56, 0.78) | | <0.001 |
| Model 2 | 1 | 0.93 (0.80, 1.08) | | 0.89 (0.77, 1.04) | | 0.72 (0.61, 0.85) | | 0.002 |
| Model 3 | 1 | 0.93 (0.80, 1.08) | | 0.91 (0.78, 1.07) | | 0.73 (0.61, 0.86) | | 0.004 |
| **Coronary heart disease** | | | | | | | | |
| Model 1 | 1 | 0.93 (0.81, 1.06) | | 0.77 (0.67, 0.89) | | 0.82 (0.71, 0.95) | | 0.001 |
| Model 2 | 1 | 0.91 (0.80, 1.05) | | 0.76 (0.66, 0.88) | | 0.81 (0.70, 0.94) | | 0.001 |
| Model 3 | 1 | 0.91 (0.79, 1.04) | | 0.76 (0.65, 0.88) | | 0.79 (0.68, 0.92) | | 0.001 |
| **Angina** | | | | | | | | |
| Model 1 | 1 | 0.91 (0.77, 1.07) | | 0.78 (0.65, 0.92) | | 0.78 (0.65, 0.93) | | 0.007 |
| Model 2 | 1 | 0.90 (0.77, 1.06) | | 0.77 (0.65, 0.92) | | 0.78 (0.65, 0.93) | | 0.008 |
| Model 3 | 1 | 0.90 (0.76, 1.06) | | 0.78 (0.66, 0.93) | | 0.78 (0.65, 0.94) | | 0.014 |
| **Heart attack** | | | | | | | | |
| Model 1 | 1 | 0.79 (0.70, 0.91) | | 0.72 (0.63, 0.83) | | 0.66 (0.57, 0.76) | | <0.001 |
| Model 2 | 1 | 0.81 (0.71, 0.93) | | 0.76 (0.66, 0.87) | | 0.70 (0.60, 0.81) | | <0.001 |
| Model 3 | 1 | 0.81 (0.71, 0.93) | | 0.77 (0.67, 0.88) | | 0.70 (0.60, 0.81) | | <0.001 |
| **Stroke** | | | | | | | | |
| Model 1 | 1 | 0.77 (0.68, 0.89) | | 0.66 (0.58, 0.77) | | 0.66 (0.57, 0.77) | | <0.001 |
| Model 2 | 1 | 0.81 (0.71, 0.93) | | 0.72 (0.62, 0.83) | | 0.72 (0.62, 0.84) | | <0.001 |
| Model 3 | 1 | 0.81 (0.71, 0.94) | | 0.73 (0.63, 0.85) | | 0.73 (0.63, 0.85) | | <0.001 |

Abbreviations: OR, Odd ratio; CI, confidence interval; Q, quartile.

Vitamin E (μg) was divided to four levels by quartile (Q1 ≤ 4.3; 4.3 < Q2 ≤ 6.9; 6.9 < Q3≤ 10.8; Q4 > 10.8).

Model 1 was adjusted as age and sex;

Model 2 was adjusted as model 1 plus race, education levels and poverty;

Model 3 was adjusted as model 2 plus smoking, drinking, BMI, total cholesterol, dietary supplement use, diabetes and hypertension.

**Supplement Table 6.** Logistic regression model to assess the association between iron and specific CVD risk.

| Specific CVD | Q1 | Q2 | Q3 | | Q4 | | *P* for trend | |
| --- | --- | --- | --- | --- | --- | --- | --- | --- |
|  | OR | OR (95% CI) | OR (95% CI) | | OR (95% CI) | |  |  |
| **Congestive heart failure** | | | | | | | | |
| Model 1 | 1 | 0.86 (0.74, 0.99) | | 0.68 (0.58, 0.80) | | 0.64 (0.55, 0.76) | | <0.001 |
| Model 2 | 1 | 0.91 (0.78, 1.06) | | 0.74 (0.63, 0.87) | | 0.70 (0.59, 0.83) | | <0.001 |
| Model 3 | 1 | 0.93 (0.80, 1.08) | | 0.75 (0.64, 0.88) | | 0.73 (0.61, 0.87) | | <0.001 |
| **Coronary heart disease** | | | | | | | | |
| Model 1 | 1 | 0.97 (0.84, 1.12) | | 0.94 (0.81, 1.08) | | 0.90 (0.77, 1.04) | | 0.517 |
| Model 2 | 1 | 0.94 (0.81, 1.09) | | 0.90 (0.78, 1.04) | | 0.84 (0.72, 0.97) | | 0.135 |
| Model 3 | 1 | 0.95 (0.82, 1.10) | | 0.91 (0.78, 1.06) | | 0.86 (0.74, 1.01) | | 0.271 |
| **Angina** | | | | | | | | |
| Model 1 | 1 | 0.85 (0.72, 1.01) | | 0.91 (0.77, 1.07) | | 0.79 (0.66, 0.94) | | 0.055 |
| Model 2 | 1 | 0.84 (0.71, 0.99) | | 0.88 (0.74, 1.05) | | 0.75 (0.62, 0.90) | | 0.017 |
| Model 3 | 1 | 0.85 (0.72, 1.01) | | 0.90 (0.76, 1.07) | | 0.78 (0.65, 0.94) | | 0.056 |
| **Heart attack** | | | | | | | | |
| Model 1 | 1 | 0.79 (0.69, 0.91) | | 0.83 (0.73, 0.96) | | 0.70 (0.61, 0.81) | | <0.001 |
| Model 2 | 1 | 0.81 (0.71, 0.94) | | 0.86 (0.75, 0.99) | | 0.72 (0.62, 0.83) | | <0.001 |
| Model 3 | 1 | 0.83 (0.72, 0.95) | | 0.89 (0.77, 1.02) | | 0.75 (0.64, 0.87) | | 0.001 |
| **Stroke** | | | | | | | | |
| Model 1 | 1 | 0.75 (0.65, 0.85) | | 0.65 (0.57, 0.75) | | 0.57 (0.49, 0.67) | | <0.001 |
| Model 2 | 1 | 0.79 (0.69, 0.91) | | 0.71 (0.61, 0.82) | | 0.63 (0.54, 0.74) | | <0.001 |
| Model 3 | 1 | 0.81 (0.70, 0.93) | | 0.72 (0.63, 0.84) | | 0.65 (0.55, 0.76) | | <0.001 |

Abbreviations: OR, Odd ratio; CI, confidence interval; Q, quartile.

Iron (μg) was divided to four levels by quartile (Q1 ≤ 9.6; 9.6 < Q2 ≤ 13.2; 13.2 < Q3≤ 18.0; Q4 > 18.0).

Model 1 was adjusted as age and sex;

Model 2 was adjusted as model 1 plus race, education levels and poverty;

Model 3 was adjusted as model 2 plus smoking, drinking, BMI, total cholesterol, dietary supplement use, diabetes and hypertension.
